# RUBIC: An Untethered Soft Robot With Discrete Path Following

**DOI:** 10.3389/frobt.2019.00052

**Published:** 2019-07-12

**Authors:** Hsing-Yu Chen, Richard Suphapol Diteesawat, Alice Haynes, Alixander James Partridge, Melanie Florine Simons, Enrico Werner, Martin Garrad, Jonathan Rossiter, Andrew T. Conn

**Affiliations:** ^1^Bristol Robotics Laboratory, University of Bristol, Bristol, United Kingdom; ^2^Department of Mechanical Engineering, University of Bristol, Bristol, United Kingdom; ^3^EPSRC Centre for Doctoral Training in Robotics and Autonomous Systems (FARSCOPE), University of Bristol and University of the West of England, Bristol, United Kingdom; ^4^Department of Engineering Mathematics, University of Bristol, Bristol, United Kingdom

**Keywords:** soft robotics, locomotion, untethered, fluidic elastomer actuators, RoboSoft

## Abstract

Soft robots have the potential to diminish the need for humans to venture into unsuitable environments or work in extreme conditions. While their soft nature gives them the advantage of being adaptable to changing environments, their control can be challenging because of the compliance that makes them effective. In this paper we present RUBIC: the Rolling, Untethered, Ballooning, Intelligent Cube, that overcomes some of the difficulties of 2D control by constraining motion to a discretised Cartesian space. RUBIC's method of locomotion is by rolling from one face of the cube to another, in any one of four directions. This motion causes it to move within a 2D grid structure, the dimensions of which are defined by the cube's characteristic length. When in its resting position RUBIC is inherently stable and forms a safe platform for tasks including taking measurements and soil samples, for localization and *ad hoc* network infrastructure, and as the foundation for larger robots and structures. We present the design of RUBIC's body, the four pneumatic ballooning actuators per face that generate its unique gait, and the control systems for locomotion and obstacle climbing. We consider constraints imposed by the design and fabrication methods including physical dimension and weight, material properties and control fidelity. An alternative locomotion scheme is proposed to improve the speed and linearity which also increases the distance traveled per roll. RUBIC travels with a mean locomotion accuracy of 4.58° deviation and successfully traverses steps up to 35% of its own height. The discretisation of a soft robotics workspace, as demonstrated by RUBIC, has advantages for safe and predictable locomotion and has applications in both structured and hazardous environments.

## 1. Introduction

Soft robotic locomotion is of interest due to the ability of compliant systems to deal with uncertain terrain. These techniques are typically inspired by biological organisms, such as caterpillars, snakes and insects (Kim et al., 2013). There are a range of different materials and soft actuators that can be used for the fabrication of such devices, such as elastomers (Marchese et al., 2015), shape memory alloys (SMAs) (Umedachi et al., 2016), dielectric elastomers (Li et al., 2018), and kirigami skins (Rafsanjani et al., 2018).

Of these materials, elastomers are a popular choice as they are highly versatile and compliant whilst being light-weight (Ilievski et al., 2011). For these reasons, they have been used in a variety of locomoting soft robots. One such soft robot designed by Shepherd et al. (2011) and Tolley et al. (2014a) demonstrates a crawling motion. The soft robot is completely manufactured with the elastomer silicone and uses this material to create a frictional difference with the ground between the leading and tail end of the robot that alternates as it moves to favor forward motion. However, the frictional contact between the elastomer and the ground leads to a high rate of wear, in turn causing a risk of puncture. This limitation can also be seen in the robot that uses vibrations to bounce across a surface on its elastomer bellows (Kühnel et al., 2016). The walking legged robots by Nemiroski et al. (2017) utilize elastomers to create joints to move more rigid components or legs. These are potentially better suited to traversing uneven terrains than crawling robots, not only as the soft elastomer is less likely to get damaged as it is not in contact with the surface, but also because they have a smaller surface area in contact with the ground, resulting in less energy being used to overcome friction and allowing them to navigate over obstacles (Siegwart and Nourbakhsh, 2004).

Rolling has the potential to be a faster method of locomotion for soft robots, however it comes at a cost of increased unpredictable path following and complex simulation and control (Li et al., 2018). Different technologies have been used to achieve the rolling motion. Li et al. (2018) created a soft robot with patterns of dielectric elastomers (DEAs) in the shape of a ring. Activating these DEAs results in extension of those regions and therefore deformation of the ring. Other methods of rolling have been achieved with pressurizing fabric tubes which have been constrained in certain directions to generate bending (Wang et al., 2019). Successful application of elastomers for the use of rolling locomotion has been demonstrated by Steltz et al. (2009) and Steltz et al. (2010) which uses a technique known as particle jamming. Silicone elastomer filled with particles and arranged in a sphere can be vacuumed to change its stiffness. Rolling is achieved when specific elastomer cells are unjammed causing them to reduce in stiffness. A central actuator then causes expansion of these cells and a change of shape of the sphere. Although a completely soft locomoting robot is achieved, the thickness of the elastomer and number of elastomer cells will limit the complete morphing ability of the robot.

Applications of soft robotics for locomotion are expansive and have overcome some of the challenges faced by rigid robots. However, their pliable nature comes with the challenge of control due to their high number of degrees of freedom. The non-linearities within the actuation of soft robots can result in systems that are difficult to predict and the repeatability of motion can be challenging. As such, control schemes for soft robotic systems often require intrinsic and extrinsic sensing and/or localization, alongside complex control systems for locomotion. Additionally, current systems generally lack the stability of conventional robotic systems and resting positions can vary from step to step (Tolley et al., 2014b). Soft robotic systems often also require tethering to off-board pneumatic and electronic components, resulting in long tethers that limit the robot's range of motion and reach (Shepherd et al., 2011).

**Figure 1:**
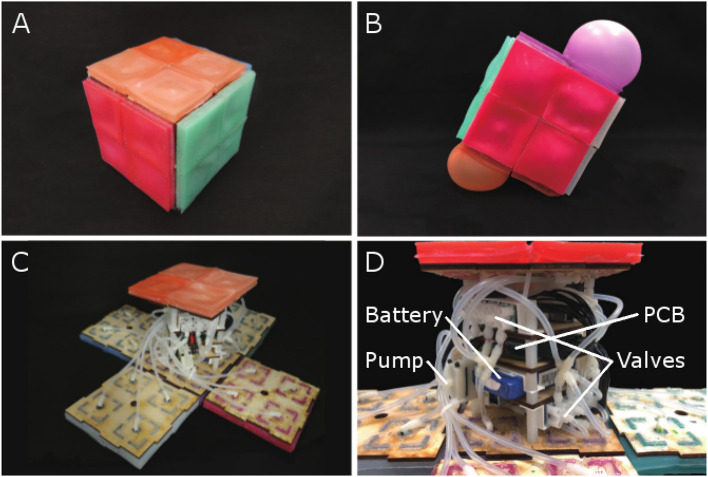
**(A)** RUBIC, stationary. **(B)** Demonstration of untethered rolling via pneumatic actuators. **(C)** RUBIC with open casing. **(D)** The internal structure of RUBIC.

We present RUBIC, the Rolling, Untethered, Ballooning, Intelligent Cube. The external structure, actuation mechanisms and internal structure are shown in Figure 1. RUBIC utilizes fluidic elastomer actuators to locomote from face to face, as shown in Figure 1B. In contrast to past soft robotic systems, RUBIC can approximately follow a discrete, predictable path along a grid. In addition, RUBIC is operable untethered as all electronics are on-board and commands are received via remote control. In this paper we describe the design of RUBIC, with focus on the characterization of the actuators and quantification of locomoting patterns.

## 2. Robot Design

The fundamental concept of RUBIC is a cube that locomotes by rolling. Actuation is provided by 24 fluidic elastomer actuators, attached to the faces of the cube, that inflate to create a rolling motion, as shown in Figure 1B. To achieve locomotion in two dimensions, each face comprises four actuators in a 2 by 2 matrix. When two adjacent actuators on the bottom face of the cube inflate, they lift that side of the cube causing it to roll onto the adjacent face. Repeating this process allows RUBIC to navigate through its environment along a discretised path. The cubic structure ensures that the robot can be directed left, right, forwards or backwards with a simple control system. Power is provided via an on-board lithium polymer battery, allowing for approximately an hour of locomotion per charge. Control signals are sent via Bluetooth, allowing the robot to operate untethered.

A cube was selected as the base structure for the robot based on the outcome of analysis into the dynamics of platonic solids with equal volume. The five platonic solids were considered: tetrahedron, cube, octahedron, dodecahedron and icosahedron. These solids were then analyzed based on three factors: rotation angle; actuator volume and energy. For rotation angle α, we calculated the angle each solid must be rotated from its stationary position in order to roll to the next face, e.g., 45° for a cube.

(1) $$\begin{array}{r} \alpha =90-\arcsin\left(\frac{r_{in}}{r_{mid}}\right) \end{array}$$

where *r*_*in*_ and *r*_*mid*_ are the inradius and midradius of the platonic solid, respectively. For actuator volume *V*_*actuator*_, we modeled the fluidic elastomer actuators as spherical caps, such that we could calculate the volume they need to inflate to in order for the solid to rotate to the rotation angle.

(2) $$\begin{array}{r} V_{actuator}=V_{sphere}-V_{cap}=\frac{4}{3}\pi R^{3}-\frac{1}{6}\pi h\left(3{\left(\frac{c}{2}\right)}^{2}+h^{2}\right) \end{array}$$

with sphere volume *V*_*sphere*_, spherical cap volume *V*_*cap*_, sphere radius *R*, spherical cap height *h*, and actuator diameter *c*. For energy, we calculated the kinetic energy by comparing the potential energy in the resting position to the potential energy when rotated to the rotation angle.

(3) $$\begin{array}{r} KE=mg\left(H^{{}^{\prime }}-H\right)=mg\left(r_{mid}-r_{in}\right) \end{array}$$

where *H* and *H*′ are the height of the robot at resting and the turning position, respectively. For further details please see the Supplementary Materials.

**Figure 2:**
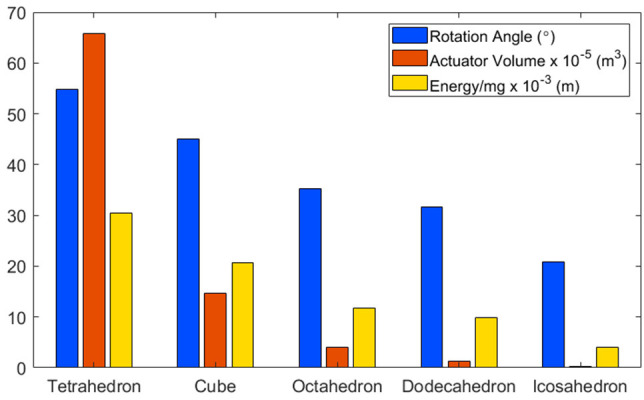
A comparison of platonic solids, based on: (i) Rotation Angle: The angle each solid must be rotated in order to flip to the next face. (ii) Actuator Volume: The volume an actuator would have to inflate to in order to reach the rotation angle. (iii) Energy: The potential energy required to reach the rotation angle.

The outcome of this analysis is that rotation angle, actuator volume and energy decrease with increasing number of faces, as shown in Figure 2. However, there is an exponential decrease in actuator volume. This indicates that tetrahedrons would require actuators to inflate to over four times larger than those actuating a cube.

Though icosahedron have the lowest values in all three metrics, there are other factors to consider. Fabrication complexity increases with the number of faces, as each face requires between 3 and 5 actuators, depending on the solid. Thus, cubes and octahedrons would require 24 actuators to enable multi-directional locomotion and dodecahedrons and icosahedrons would require 60. Another consideration is in the solid's stability when at rest, as it is anticipated that the resultant robot would be used in unstable environments. In this instance, a higher rotation angle is beneficial, as it means that the solid is less likely to roll due to environmental perturbations. As such, cubes were selected based on the low actuator volume required to reach the rotation angle compared with tetrahedrons and their stability compared with solids with more faces. Additionally, cubes are the only platonic solid capable of straight line locomotion, as all other solids follow angular paths when rolled from face to face.

### 2.1. Robot Fabrication

#### 2.1.1. Internal Structure

**Figure 3:**
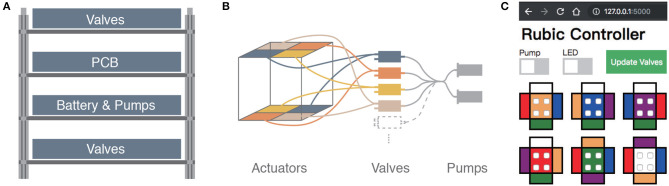
**(A)** Schematic of layered structure inside RUBIC to house the battery, PCB and pneumatic system. **(B)** Schematic of pneumatic system for two of the six sides of RUBIC, from pumps (far right) to pairs of actuators via the valves. **(C)** Screenshot of the GUI used to control RUBIC.

The internal structure of RUBIC can be seen in Figures 1D, 3. We designed the layout to allow for an even distribution of weight, thereby minimizing the interference with the kinematics of the cube. The components are fixed to 4 layers of laser cut plywood as shown in Figure 3A. The outer two layers house 12 3-way solenoid valves (5-6 V; Zonhen Electric Appliances), 6 on the top layer and 6 on the base layer. The middle two layers contain the PCB, the Lithium-ion Polymer battery (7.4 V; 1000 mAh, Turnigy) and 2 pumps (3 V, KPM14A; Koge Electronics CO., LTD.). Both pumps are connected to all 12 valves to ensure maximum pneumatic power, and each valve is connected to two actuators on opposite sides of the cube, as shown in Figures 1D, 3B.

A key challenge in our design of RUBIC was to minimize size and weight in order to reduce the load on the pumps and increase locomotion speed. Size was a particular issue as actuating a larger cube would require a greater torque and create a greater distance for the actuators to lift in order to locomote. As a compromise, we chose to use 12 valves connected to opposing pairs of actuators, rather than having 24 valves with one valve per actuator. This saved on space and weight, allowing RUBIC to be a 10 cm cube and 0.83 kg, while containing all of the required electronics. However, this resulted in a decrease in speed since the pumps were then required to pump twice as many actuators at a time. It is also the cause of RUBIC's unique appearance when locomoting, observable in Figure 1B.

#### 2.1.2. Actuators

**Figure 4:**
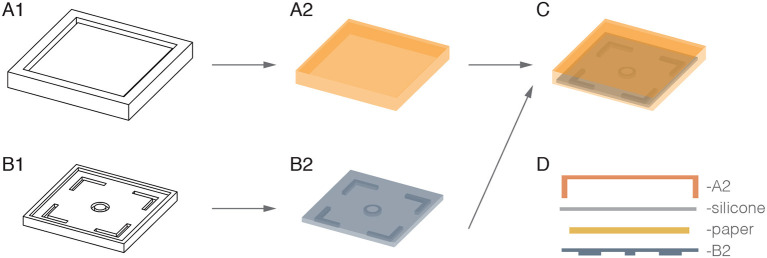
Illustration of actuator fabrication in a 2-step molding process. The molds **(A1,B1)** were used to separately fabricate the silicone covers **(A2)** and bases **(B2)**. These were then cured together with another layer of silicone, into which a piece of paper was placed to create a complete air chamber with a radially constrained base **(C,D)**.

We manufactured the pneumatic actuators from silicone elastomer (Ecoflex 00-30^*TM*^; Smooth-On), as it is light-weight, versatile and highly compliant, reaching up to 900% expansion before tearing (Smooth-On, 2018). Further characterization of the elastomer has been performed by Sparks et al. (2015). Actuators were manufactured in a 2-step molding process. Bases and covers were fabricated separately, before being cured together along their edges to create a sealed air chamber, as shown in Figure 4. Each actuator measures 50 × 50 × 8 mm. The wall and ceiling thicknesses of each actuator are approximately 2 mm and 1.5 mm respectively. The base of the actuators are 3 mm thick and contain a constraining paper layer to limit expansion at the base (Figure 4D).

We designed the actuators with four right angled silicone extrusions to ease connection with the internal Medium-Density Fiberboard (MDF) body. A ring of silicone was also extruded from the center of the actuator to interface with the pneumatic input (external and internal diameters of 8 mm and 4 mm, respectively). Extruded structures are 4 mm in length, long enough to extend through holes in the 3 mm MDF body. Actuators were fixed to the internal structure of the cube with the use of silicone adhesive (Sil-Poxy^*TM*^; Smooth-On). We dyed the actuators a different color for each side of the cube to make them distinguishable and to simplify setting up the control mechanism.

### 2.2. Principle of Operation

At start up, the Bluetooth communication channel is enabled (visual feedback is provided by an LED to confirm connection) and all valves and pumps are off. We control the robot from a PC or tablet via a graphical user interface (GUI) that was developed for ease of operation, as shown in Figure 3C. The GUI includes switches to initialize pumps and manipulate which valves are open or closed at any time. We update valves by selecting the actuator on each image that needs to be opened and then click “Update Valves,” thus updating all valves at once. Opening a single valve translates to activating two actuators on the robot, as described in section 2.1.1 and illustrated in Figure 3B. We operate the robot by observing which side faces the ground, finding the corresponding colored face within the GUI and opening the two valves opposite to the direction of travel. The GUI updates all valves simultaneously, overcoming potential problems with off-setting actuation, which would result in an unwanted tilt.

We designed RUBIC to discretise the operable space surrounding it. This is a novelty that can be difficult to accomplish with soft robotic components, due to non-linearity of actuation and the high number of degrees of freedom that soft materials exhibit. In principle, this means that RUBIC translates its environment into a grid structure along which it locomotes. Inflating two actuators opposite to the direction of travel results in locomotion to the next grid point, either to the left, right, front or back. This discretisation allows for predictions to be made about how RUBIC can move in space. It also allows environments to be mapped such that routes can be planned prior to actuation of the robot.

## 3. Actuator Characterization

The fluidic elastomer actuators are the fundamental components that allow locomotion of this robot. In this section we detail the steps taken to characterize these actuators.

### 3.1. Method

We designed RUBIC to operate untethered, thus increasing its operation space and eradicating restrictions resultant from a tethered connection. In order to operate untethered, all of the components had to be integrated inside the body of the robot. To save space, smaller pumps had to be used and the number of valves reduced, as shown in Figure 3 and discussed in section 2.1.1. Consequently, the pumps inflate two actuators simultaneously. To investigate the performance impact of this set-up we tested the actuators in terms of their vertical displacement and pressure reached with time for actuation of single and double (paired) actuators. We conducted tests with pumps equivalent to the on board pumps installed in RUBIC. Testing in this way allowed for a comparison in terms of both speed and pressure dynamics.

We attached the actuators to the bottom and, in the double actuator test, to the top of a linear guide. To test the performance of a single base actuator in lifting the robot, we set the weight of the supporting structure to match half of the robot's weight, as we assumed that one actuator provides half of the lift required for locomotion. We inflated the actuators until a height of 75 mm was reached, as this was the approximate height required to roll onto the next face, described in section 4.1 (**Figure 7**). We maintained this level until a valve (S070C-SBG-32; BEST Pneumatics Inc.) was opened to deflate the actuators. We measured the height with a laser displacement meter (LK-G152; Keyence) and the pressure inside the actuators with a pressure gauge (HSCDANN030PGAA5; Honeywell). The results we obtained allow for comparison between the single (unpaired) and double (paired) actuators.

### 3.2. Results

**Figure 5:**
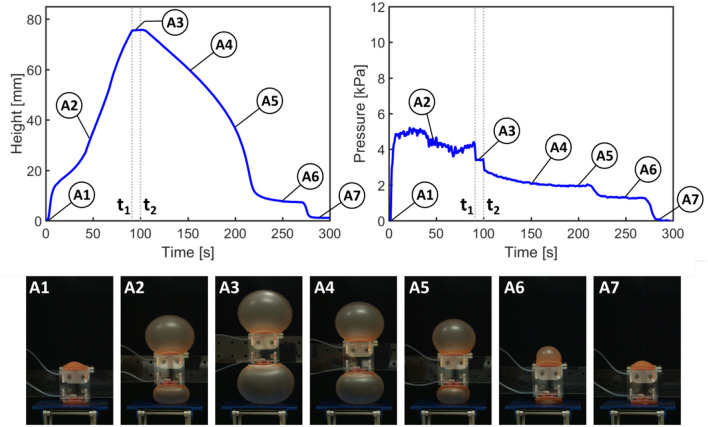
Results of height and pressure measurements for actuation of paired actuators (as in RUBIC). *t*_1_ signifies the time at which the pump is switched off; *t*_2_ signifies the opening of the valve to release the air.

**Figure 6:**
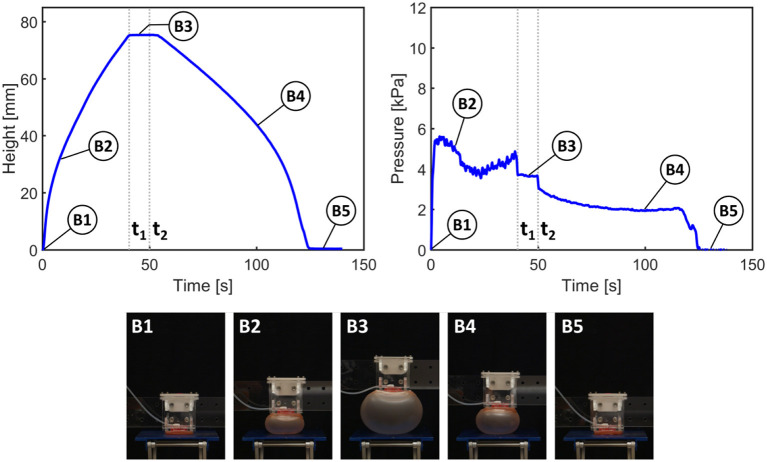
Results of height and pressure measurement for actuation of a single actuator. *t*_1_signifies the time at which the pump is switched off; *t*_2_ signifies the opening of the valve to release the air.

In Figures 5, 6, from time 0 to *t*_1_, the pump is switched on to activate inflation of the actuators. From *t*_1_ to *t*_2_, the pump is switched off but the air is maintained in the system (i.e., the actuators are kept inflated); after time *t*_2_ the valve is opened to release the air and allow the actuators to deflate.

Figure 5 shows the height and pressure measurements for actuation of the paired actuators, closely representing the actuation scheme used in RUBIC. For each pair of actuators, only the actuator on the bottom face of RUBIC is load-bearing and responsible for lifting the robot. To replicate this in the paired actuator experiment, we applied a load to one actuator and allowed the other to actuate freely. During the initial inflation period, the rate of vertical displacement drops temporarily (between A1 and A2) because the inflation of the top actuator is greater than that of the bottom actuator. As the pressure remains constant, this behavior can be explained by the slight difference in material thickness of the actuators and the additional external load acting on the bottom actuator (i.e., the weight of the test rig). While maintaining constant height, the actuator rapidly snapped through to a stable, high-strain ballooned state. This dynamic snap-through is a well studied phenomena with inflated hyperelastic membranes (Akkas, 1978). Toward the end of the deflation period (A6), the rate of pressure and height change levels due to a fold that developed in the bottom actuator. In that period, the top actuator deflated at a higher rate before both actuators returned to their initial configuration (A7).

The same general behavior can be observed using only one actuator. However, the actuator inflates at a steadier rate (B1 and B2 in Figure 6) as no mutual interference between the two actuators occurs. It also takes approximately half the time to reach the desired height. Considering the same general behavior for the two tested actuator configurations, the use of double actuator inflation is a valid trade-off to enable an untethered robot, although at the cost of locomotion speed.

## 4. Locomotion Characterization

Locomotion of RUBIC is achieved by rolling from one face onto the next in a quantized Cartesian space. In this section, we describe the steps taken to characterize the locomotion abilities of RUBIC.

### 4.1. Analysis of Locomotion Schemes

#### 4.1.1. Method

**Figure 7:**
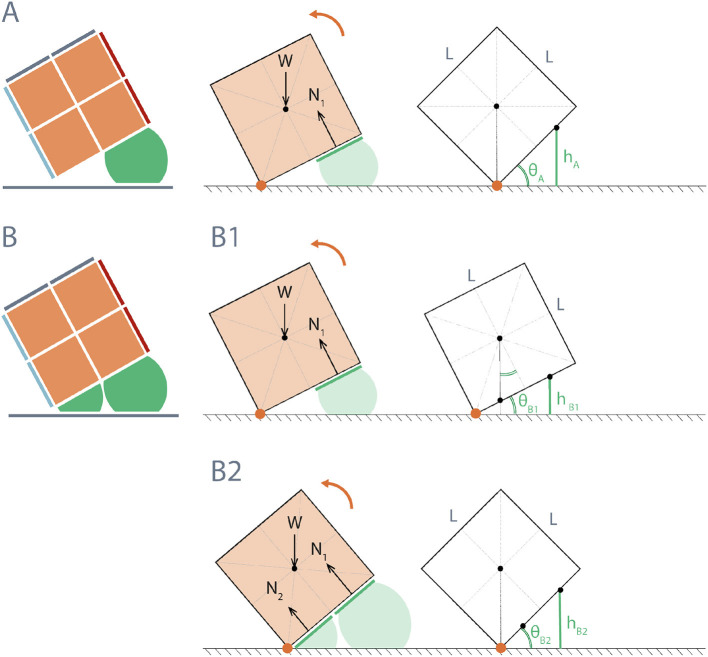
Diagram of height required from actuators to reach tipping angle of RUBIC for two actuation schemes: **(A)** Rear actuators only; θ_*A*_ = 45°, resulting in actuator height *h*_*A*_ = 3Lsinθ_*A*_/4 = 53.03 mm for *L* = 100 mm. **(B)** Front and rear actuators; **(B1)** rear actuators initially activated until RUBIC reaches minimum angle $\theta_{B1}=tan^{-1}\left(1/2\right)=26.6$°, where the center of mass shifts beyond the center of the front actuators (*L*/4 from hinge point), *h*_*B*1_ = 33.58 mm for *L* = 100 mm, **(B2)** front actuators are then additionally activated until tipping angle θ_*B*2_ = 45° is reached, (where *h*_*B*2_ = *h*_*A*_).

Locomotion of RUBIC consists of opening pairs of valves, allowing time for the actuators to fill with air and waiting for a tipping point to be passed (the moment at which the center of mass of the robot passes over the point of contact with the ground). A single step of the robot can be modeled simply by considering the leading edge of RUBIC as a hinge and then calculating the diameter the actuator has to inflate to (inflated diameter) in order to reach the tipping point, as depicted in Figure 7A. Assuming uniform weight distribution within the robot, we deduce that the angle required for the robot to reach its tipping point is 45° when the robot locomotes with rear actuators alone. We propose that a second locomotion pattern could reduce the time required to complete a single step. This second pattern consists of activating the rear actuators as before, but then activating the front actuators during the roll to assist the motion.

Figure 7B illustrates a theoretical model for this actuation pattern, again assuming uniform weight distribution. For the front actuators to assist, not impede, the roll, they must be activated once the center of mass has passed over the center of the front actuator. In doing so they provide additional torque about the hinge point. Activation prior to this point does not assist with locomotion, where the angle θ at which the front actuators should be activated was calculated from the geometry of a cube to be at least 26.6° as shown in Figure 7B1.

**Figure 8:**
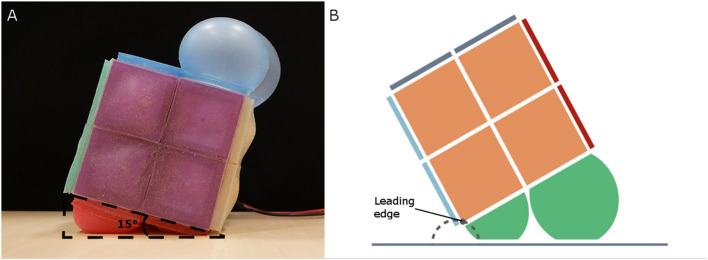
**(A)** Test set-up for alternative actuation pattern, showing threshold angle (θ = 15° in this case). **(B)** Approximate leading edge trajectory (dashed gray) of RUBIC when alternative actuation pattern is used.

To test this alternative actuation pattern, we placed RUBIC in front of a blackout curtain with space to locomote forwards. We cut several freestanding plywood triangles to act as physical representations for each value of angle θ being tested and placed these in front of RUBIC. We then inflated the rear actuators until the base of RUBIC matched the freestanding representation of θ, at which point we inflated the front actuators as shown in Figure 8A. We performed three tests for θ = 10°, 15°, 20°, and 25°. We then extracted time-stamped data, including the point at which valves are opened, the point at which the front actuators are actuated and the end of the roll, from recorded video and used this data to evaluate the effect of changing angle θ.

To determine the linearity of RUBIC's locomotion, we measured the angle of deviation from a straight line path. A primary novelty for RUBIC is the quantization of its environment and its movement within a 2D grid. In order for this pattern of locomotion to be achievable and predictable, the path that RUBIC follows when rolling in any one direction must be as straight as possible.

**Figure 9:**
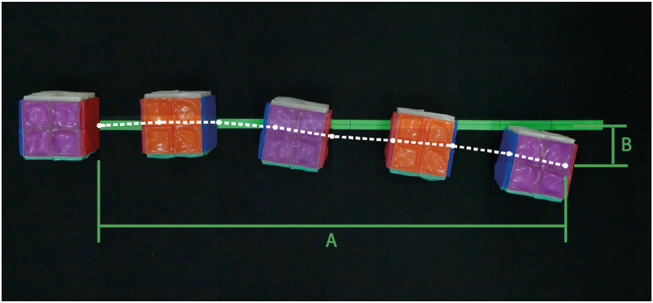
Example deviation of RUBIC from the central line during the course of 2 full rotations using the alternative actuation scheme (front and rear actuators used). Measured trajectory of leading edge shown in white. Final displacement **(A)** = 909.75 mm and deviation from center **(B)** = 78.51 mm.

We aligned the robot to a straight line on a large, flat surface and recorded its starting position. RUBIC then locomoted step by step along this line until two full rotations were complete (resulting in a total of 8 steps being taken). We then recorded the final position and calculated the angle of deviation. We ran 5 trials for both the original locomotion pattern (using rear actuators only) and the alternative pattern (using both rear and front actuators, with front actuators activated once RUBIC reached an angle of 15°, based on the results of the previous test). We also recorded position data for each step within two trials of the alternative actuation pattern to illustrate an example linear path. Figure 9 shows the experimental set-up and an example path for the line-following experiment where lengths A and B equate to the measured distance and deviation of the final position relative to the central line. We calculated the overall deviation angle α as α = *arctan*(*B*/*A*).

**Figure 10:**
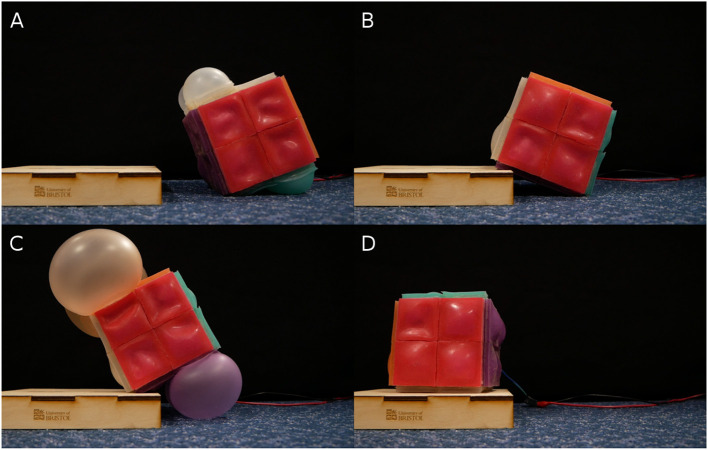
Snapshots of RUBIC traversing up a 35 mm step in chronological order from **(A–D)**.

To evaluate RUBIC's capabilities over uneven terrain, we devised a test to measure its performance traversing a surface that included steps that ranged from 20 to 40 mm in 5 mm increments. For each step, we aligned RUBIC such that the robot landed with the center of the robot just off the edge of the step, as shown in Figure 10B. In this way, the rear actuators were not on the step and were able to continue pushing the ground. The front actuators were then actuated once the base of the robot had reached a 15° angle with the step, making contact with the step and assisting the roll. It was expected that RUBIC would not be able to traverse a step greater than 50 mm since the pivot point between RUBIC and the step would be too high to lift the center of mass over. Video was taken of the experiment and the set up can be seen in Figure 10. We extracted time data from video recordings as a metric to compare different climbing methodologies.

#### 4.1.2. Results

For the original locomotion scheme, Figure 7A, with only rear actuators activated, the angle required to make a step was calculated to be 45°. In practice, this was successful and one roll was found to take 55.03 s with standard deviation of 1.04 s across 3 trials.

The most time efficient control plan for activating the front actuators was at θ = 15°, decreasing the total time taken to roll to 51.70 s. Activating the front actuators at an angle of 10° failed, resulting in lifting the front side of RUBIC upward instead of assisting the roll. Additionally, it was found that actuation of the front actuators at θ = 15° increased the total step length by 1.10% as listed in Table 1. This occurred due to the inflation of the front actuators generating both a turning force in the direction of travel and an upwards force that lifted the leading edge of the cube from the ground. This has the effect of shifting the point of turn beyond the edge of the cube, resulting in an extended step length. The trajectory of the leading edge in this scenario is shown in Figure 8B.

**Table 1:** Mean deviation of RUBIC from the central line after two full rotations (eight individual rolls) across five trials for the original locomotion scheme and for the alternative scheme.

**Locomotion scheme**	**Mean deviation (mm)**	**Mean distance (mm)**	**Mean angle (**°**)**	**SD of angle (**°**)**
Original: rear actuator only	110.8	909.7	7.09	3.24
Alternative: front actuators activated at 15°	72.8	919.7	4.58	2.78

*Angle calculated from deviation and total distance travelled*.

Figure 9 gives an example trajectory of RUBIC as it traversed along the central line. As can be seen, the deviation of RUBIC from the central line increases with each roll. The total deviation and distance traveled by RUBIC were measured and the mean results can be seen in Table 1. The results show that the alternative locomotion scheme, while being faster than the original scheme as previously discussed, also results in a smaller deviation of RUBIC from the central line as it travels. Not only this, but the overall distance traveled by RUBIC for the two rotations was 10 mm further than in the rear actuator case.

The deviation of RUBIC from the central line was 12.18% of the distance traveled for the original locomotion scheme and 7.92% for the alternative scheme. While the alternative locomotion scheme (front and rear actuators) is faster and more precise than the original locomotion scheme (rear actuators only), the results of this test show that there is uncertainty in the discretised space RUBIC can navigate. The further RUBIC travels, the greater the uncertainty of its final position. Observation of video recordings shows that much of this uncertainty is due to irregularities in the fabrication of the actuators, as actuators will inflate to different sizes at different rates depending on the thickness of the actuator walls. This will be elaborated on in the discussion.

RUBIC was successfully able to climb step sizes of up to 35 mm, but was not capable of climbing a 40 mm step. The failure of RUBIC to navigate the 40 mm step was due to the maximum expansion of the actuators. However, completion of a 35 mm step is over a third of RUBIC's height and future iterations of RUBIC can be scaled according to the demands of the environment. In addition, less dramatic terrains with slopes and bumps, rather than sharp corners and steps, may be less of a challenge for RUBIC.

## 5. Discussion

In this paper, we have demonstrated RUBIC's ability to locomote on a flat surface and across uneven terrain. We have also characterized the fluidic elastomer actuators and provided two differing locomoting patterns for RUBIC.

For the alternative locomotion scheme, an angle of 26.6° was calculated to be the optimal angle for actuation of the front actuators as illustrated in Figure 7B. However, in practice, the optimal angle was 15° which reduced the time per roll by 3.33 s. Two reasons are presented for this discrepancy between the theoretical and practical optimal angle. Firstly, when the front actuators are activated there is a short delay while they inflate before they touch the ground and provide active assistance. During this time, RUBIC continues to rotate and, therefore, the angle at which the front actuators are providing assistance is greater than the angle at which they are initially activated. Secondly, the theory does not take into consideration the added torque of the actuators inflating and pushing against each other. When the front actuators inflate they are enclosed by RUBIC and the inflated rear actuators, and so inflation generates a force against both. This force against the rear actuators would add to the torque acting to roll RUBIC.

Deviation from a straight line path while locomoting was also reduced with the alternative locomotion scheme, as shown in Table 1. The angle of deviation was reduced by 2.51° for two complete rotations (i.e., 8 individual rolls), resulting in a linear deviation of 7.92% of the distance traveled. In addition, the distance traveled was increased with the alternative scheme by 1.10% for two complete rotations. The reason for this being that the turning point is shifted forwards (in the direction of travel) during actuation, as actuating the front actuators raises the leading edge (previously the pivot point) of the robot from the ground as illustrated in Figure 8B.

We initially proposed the alternative locomotion scheme to improve RUBIC's locomotion speed. Though testing shows that only 3.33 s are saved per roll, a 6% improvement, the alternative locomotion scheme offers other benefits, such as stair climbing, as well. While discrete movement in space is beneficial for route planning and allowing for prediction of motion, there may be instances when RUBIC becomes stuck, or unable to take a full step in a specific direction and needs to locomote away from its grid path. An instance of this could be if RUBIC is moving within a narrow corridor and its movement grid is misaligned, such that operating in a straight line would result in hitting either wall. In this instance, it would be beneficial to be able to realign RUBIC's locomotion grid, such that RUBIC can then navigate the corridor. Alternating between the alternative locomotion scheme and the original locomotion scheme provides one such method for realigning RUBIC's grid space, as each locomotion scheme has a different step length. This means that small adjustments can be made to RUBIC's locomotion to allow for such difficulties.

One of the major drawbacks in this iteration of RUBIC is the pairing of actuators. Due to the coupling of actuators to a single valve, if the power of the pumps is insufficient, the top, unloaded actuators inflate first until they reach the back pressure of the pumps (i.e., when the pressure in the actuator is equal to the pressure from the pumps) only after which the bottom loaded actuators will start to be inflated. The redundant actuation of the top actuators slows the speed down. Actuating a single actuator at a time, rather than two simultaneously as seen in RUBIC, significantly reduced inflation times and would, therefore, increase locomotion speed. However, due to limited internal space, a larger pump or more valves (to allow one valve per actuator) was not practical in this iteration of RUBIC. Future work will address this issue.

The biggest factor that limits the accuracy of the robot is irregularities in the fabrication of the actuators. Slight deviations in actuator wall thickness result in differences in inflation rate and maximum inflation diameter. As a consequence, the actuators rolling RUBIC are slightly imbalanced causing a tilt that sends RUBIC slightly off course as it rolls. This will be improved in future by refining the actuator fabrication method, ensuring precision and consistency. As a result, this deviation will be minimized and uncertainty in path following reduced.

Scalability of RUBIC is of interest for its suitability in applications that may require a smaller robot to navigate intricate environments, or a larger robot to overcome obstacles in a specific terrain. From the calculations approximating the actuator volume required for locomotion (see section 2 or Supplementary Material), we can calculate the actuator volume required for different length scales of RUBIC. This approximation gives a cubic increase in volume for a linear increase in side length. Therefore, doubling the side length of RUBIC to 0.2 m results in an 8-fold increase in actuator volume required to locomote and tripling side length to 0.3 m corresponds to a 27-fold increase in actuator volume required. If we assume a constant flow rate to the actuators this results in a cubic increase in time taken to actuate, significantly reducing locomotion speed. To increase the size of RUBIC, higher capacity pumps would be necessary to maintain performance. Reducing the size of RUBIC does not have this problem, but requires smaller valves, pumps and electronics to physically fit into the structure. These factors are limitations to the scalability of RUBIC.

In this paper, we demonstrate RUBIC, a robot with soft actuators that locomotes by rolling. The novelty of RUBIC compared with other rolling robots, such as Li et al. (2018), is that its path can be predicted, as it moves along a quantized Cartesian grid. To simplify the control mechanism, future iterations of the robot will include an Inertial Measurement Unit (IMU) so that the robot can self-sense which face is to the ground. The GUI would then indicate which face should be actuated, allowing for operation of the robot without line of sight and, ultimately, fully autonomous operation. Although many improvements can still be made, the current design of RUBIC is able to locomote untethered and across terrain that undulates by up to 35% of RUBIC's height. These are ideal properties for robots working in unstable structures, environmental monitoring and other challenging environments. Unlike other soft, rolling robots mentioned within the introduction, RUBIC is inherently stable on all of its faces and able to translate its environment to a grid space. As such, we propose future applications in environmental sampling, localization and *ad hoc* network infrastructure, or as a foundation for larger robots and structures. Future work will also explore miniaturizing the internal components and utilizing recent soft pumps and valves (Rothemund et al., 2018; Cao et al., 2019; Mahon et al., 2019) to allow for fully collapsible, untethered robots that can be readily deployed.

## Data Availability

Data are available at the University of Bristol data repository, data.bris, at https://doi.org/10.5523/bris.3dxxbzlym53ne2ou9vcjn6if3i.

## Author Contributions

H-YC, RD, AH, AP, MS, and EW: contributed equally in the design of the robot and writing of the manuscript. MG, JR, and AC: provided guidance throughout the robot design and reviewed the written content. All authors read and approved the submitted version.

### Conflict of Interest Statement

The authors declare that the research was conducted in the absence of any commercial or financial relationships that could be construed as a potential conflict of interest.
