# Supplementary material for: RUBIC: An Untethered Soft Robot With Discrete Path Following
Source: Front Robot AI. 2019 Jul 12;6:52. doi: 10.3389/frobt.2019.00052 (PMC7805893; doi:10.3389/frobt.2019.00052)
Supplement: Supplementary file 1 [file Data_Sheet_1.PDF]

Supplementary Material: RUBIC: An Untethered Soft Robot with Discrete Path Following

1 SUPPLEMENTARY TABLES AND FIGURES

Further to the work of the main text, we have chosen to model solids with alternate geometries so that future work can consider alternate morphologies. It also provides evidence for why we initially selected a cube for our work. For this work, we will model the platonic solids. Platonic solids are regular, convex polyhedra with equivalent faces of regular polygons, i.e. tetrahedron, cubes, octahedron, dodecahedron and icosahedron as illustrated in Figure S1. Throughout modelling we used platonic solids of equal volume and mass.

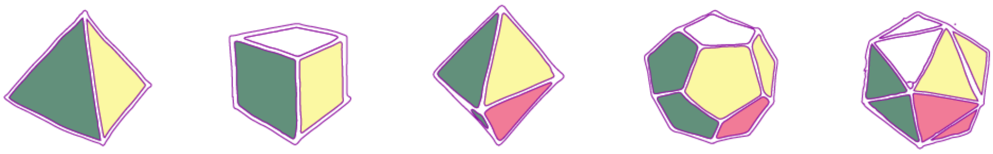

Figure S1. Platonic Solids, such as tetrahedron, cube, octahedron, dodecahedron and icosahedron (from left to right) with the number of faces of 4, 6, 8, 12 and 20, respectively.

Regarding RUBIC's design, the robot is composed of a fixed structure (modelled as a cubic platonic solid) with four soft fluidic actuators on each face, which inflate to perform movement. Locomotion can be achieved by inflating actuators on the bottom face to roll the robot over one of its edges in the desired direction, as demonstrated in Figure S4. The rotation angle and the actuator volume are used to analyse the optimal robot design.

The insphere and midsphere, tangent to the centre of each face and to the midpoint of each edge, respectively, are concentric for all platonic solids and thus can be used for calculating the rotation angle. The inradius r_{in} and midradius r_{mid} are the radii of the insphere and midsphere, respectively, which are proportional to the edge length of a polyhedron's surfaces, listed in Table S1.

Platonic Solids	Face Shape	Number of Faces, N	Inradius, r_{in}	Midradius, r_{mid}
Tetrahedron	Triangle	4	0.204	0.354
Cube	Square	6	0.500	0.707
Octahedron	Triangle	8	0.408	0.500
Dodecahedron	Pentagon	12	1.114	1.309
Icosahedron	Triangle	20	0.756	0.809

Table S1. Face shape, the number of faces, the inradius and the midradius of each platonic solid in unit length.

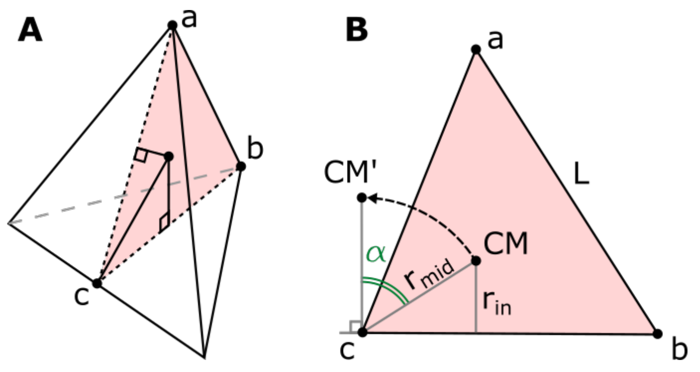

Figure S2. (A) Inradius and midradius of the tetrahedron and (B) the rotation angle related to the inradius and midradius.

The analysis model is exemplified in Figure S2 using a tetrahedron as an example. The rotation angle α can be calculated with the use of the inradius and midradius as follows.

$$\alpha = 90 - \arcsin(\frac{r_{in}}{r_{mid}}) \tag{S1}$$

Besides the rotation angle α, the distance between the turning edge and the centre of the actuated actuators, $L^{'}$ is used to calculate the actuator volume required to perform turning motion. The actuators were placed so that two actuators are always used for rolling over the turning edge, as illustrated in Figure S3.

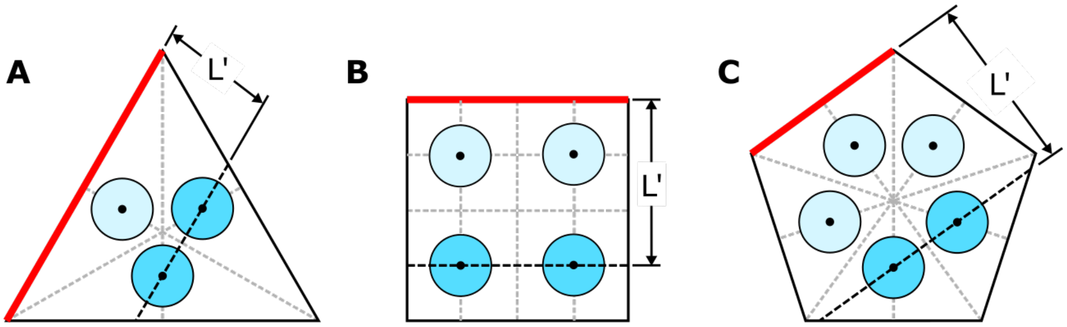

Figure S3. Allocation of the actuators (blue circles) on the bottom surface for all faces found in platonic solids: (A) triangle, (B) square and (C) pentagon, where $L^{'}$ is the distance between the turning edge (red line) and the centre of the actuators.

The actuator volume for achieving turning motion is modelled as a spherical cap as illustrated in Figure S4A (right), which can be derived with the use of the analysis model in Figure S4B as follows.

Given the rotation angle α, the distance between the turning edge and the centre of the actuated actuators $L^{'}$and the actuator diameter c, where the actuator is allocated from point A to B. Point C is tangent to the ground surface and assumes that the actuator provides sufficient friction to remain in place during actuation, and thus the distance from the turning edge to the point touching the ground, C, is equal to $L^{'}$. Let a, b and c be the length between points BC, AC and AB, respectively, thus the coordinate x and y of point A, B and C can be derived as follows.

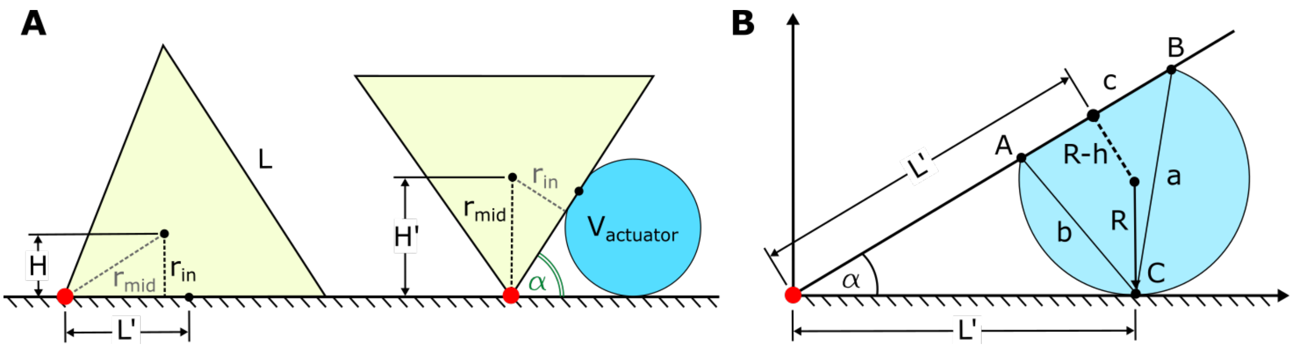

Figure S4. (A) The side view of the locomoting tetrahedron robot at the resting position (left) and the turning position (right) and (B) model of inflating actuator to calculate the required actuator volume for turning motion.

$$A = ((L^{'} - \frac{c}{2})\cos\alpha, (L^{'} - \frac{c}{2})\sin\alpha) \tag{S2}$$

$$B = ((L^{'} + \frac{c}{2})\cos\alpha, (L^{'} + \frac{c}{2})\sin\alpha) \tag{S3}$$

$$C = (L^{'}, 0) \tag{S4}$$

Therefore, the circumradius R of the inflated actuator can be calculated using the edges of the triangle ABC as follows.

$$R = \frac{abc}{\sqrt{(a+b+c)(b+c-a)(c+a-b)(a+b-c)}} \tag{S5}$$

with $R^2 = (R-h)^2 + (\frac{c}{2})^2$, then

$$h = R - \sqrt{R^2 - (\frac{c}{2})^2} \tag{S6}$$

Now, we can calculate the actuator's volume by taking the difference between the volume of a sphere and the spherical cap,

$$V_{actuator} = V_{sphere} - V_{cap} = \frac{4}{3}\pi R^3 - \frac{1}{6}\pi h(3(\frac{c}{2})^2 + h^2) \tag{S7}$$

The energy used to create locomotion can be calculated by the potential energy compared between the resting and the turning positions presented in Figure S4 as follows. Where H and $H^{'}$ are the height of the robot at the resting and turning positions, which are equal to r_{in} and r_{mid}, respectively. Therefore,

$$E_1 = E_2 \tag{S8}$$

$$KE + PE_{resting} = PE_{turning} \tag{S9}$$

$$KE = PE_{turning} - PE_{resting} \tag{S10}$$

$$KE = mg(H^{'} - H) = mg(r_{mid} - r_{in}) \tag{S11}$$

As a result, the rotation angle, the actuator volume and the energy used to achieve the turning motion for each platonic solid is presented in Table S2.

Platonic Solids	Rotation Angle (°), α	Actuator Volume ($m^3 * 10^{-5}$), V	Energy ($m * 10^{-3}$), $\frac{E}{mg}$
Tetrahedron	54.73	65.818	30.478
Cube	45.00	14.602	20.711
Octahedron	35.26	3.966	11.789
Dodecahedron	31.72	1.326	9.916
Icosahedron	20.91	0.178	4.106

Table S2. The rotation angle, the actuator volume and the energy of each platonic solid. The volume of the platonic solids are consistent at $1 * 10^{-3} m^3$ based on a cube of side length $0.1m$.
